# Prediction Model of Late Fetal Growth Restriction with Machine Learning Algorithms

**DOI:** 10.3390/life14111521

**Published:** 2024-11-20

**Authors:** Seon Ui Lee, Sae Kyung Choi, Yun Sung Jo, Jeong Ha Wie, Jae Eun Shin, Yeon Hee Kim, Kicheol Kil, Hyun Sun Ko

**Affiliations:** 1 Department of Obstetrics and Gynecology, Incheon St. Mary’s Hospital, College of Medicine, The Catholic University of Korea, Seoul 06591, Republic of Koreaobgysk@catholic.ac.kr (S.K.C.); 2 Department of Obstetrics and Gynecology, St. Vincent’s Hospital, College of Medicine, The Catholic University of Korea, Seoul 06591, Republic of Korea; eggs76@catholic.ac.kr; 3 Department of Obstetrics and Gynecology, Eunpyeong St. Mary’s Hospital, College of Medicine, The Catholic University of Korea, Seoul 06591, Republic of Korea; biondi77@hanmail.net; 4 Department of Obstetrics and Gynecology, Bucheon St. Mary’s Hospital, College of Medicine, The Catholic University of Korea, Seoul 06591, Republic of Korea; jennie1008@catholic.ac.kr; 5 Department of Obstetrics and Gynecology, Uijeongbu St. Mary’s Hospital, College of Medicine, The Catholic University of Korea, Seoul 06591, Republic of Korea; yoni@catholic.ac.kr; 6 Department of Obstetrics and Gynecology, Yeouido St. Mary’s Hospital, College of Medicine, The Catholic University of Korea, Seoul 06591, Republic of Korea; kilssine@catholic.ac.kr; 7 Department of Obstetrics and Gynecology, Seoul St. Mary’s Hospital, College of Medicine, The Catholic University of Korea, Seoul 06591, Republic of Korea

**Keywords:** fetal growth restriction, gestation, machine learning

## Abstract

Background: This study aimed to develop a clinical model to predict late-onset fetal growth restriction (FGR). Methods: This retrospective study included seven hospitals and was conducted between January 2009 and December 2020. Two sets of variables from the first trimester until 13 weeks (E1) and the early third trimester until 28 weeks (T1) were used to develop the FGR prediction models using a machine learning algorithm. The dataset was randomly divided into training and test sets (7:3 ratio). A simplified prediction model using variables with XGBoost’s embedded feature selection was developed and validated. Results: Precisely 32,301 patients met the eligibility criteria. In the prediction model for the whole cohort, the area under the curve (AUC) was 0.73 at E1 and 0.78 at T1 and the area under the precision-recall curve (AUPR) was 0.23 at E1 and 0.31 at T1 in the training set, while an AUC of 0.62 at E1 and 0.73 at T1 and an AUPR if 0.13 at E1, and 0.24 at T1 were obtained in the test set. The simplified prediction model performed similarly to the original model. Conclusions: A simplified machine learning model for predicting late FGR may be useful for evaluating individual risks in the early third trimester.

## 1. Introduction

Fetal growth restriction (FGR) is defined as not satisfactory fetal growth potential and accounts for 10% of all pregnancies [1,2]. Currently, the American College of Obstetrics and Gynecologists (2021a) and Society for Maternal-Fetal Medicine, Martins, Biggio, and Abuhamad (2020) recommend defining FGR as either an estimated fetal weight (EFW) below the 10th percentile or an abdominal circumference below the 10th percentile adjusted for gestational age [3,4].

In clinical practice, about one-third of late preterm births are related to FGR [5]. Cases with FGR have a greater risk of antenatal stillbirth, pre-eclampsia, preterm delivery, and neonatal complications such as hypoglycemia, hyperbilirubinemia, hypothermia, intraventricular hemorrhage, necrotizing enterocolitis, sepsis, respiratory distress syndrome, and neonatal death [6]. Additionally, an EFW below the 3rd percentile is specified as severe, represents a more severe form of FGR, and reportedly increases neonatal morbidity and mortality [4]. In terms of long-term outcomes, fetuses with FGR have a greater risk of neurological and cognitive disorders and metabolic diseases in adulthood [7].

FGR is subdivided into early- and late-onset based on 34 weeks of gestation [8,9,10]. Although early-onset FGR has a stronger correlation with maternal pre-eclampsia and tends to be more severe [11,12,13], late FGR is also related to fetal distress, neonatal acidosis, and cesarean section, resulting in neonatal intensive care unit admission [14]. Spectroscopy revealed that infants with late FGR had differences in levels of brain metabolites compared to normally growing infants, which were later associated with neurodevelopmental challenges [15,16].

A previous study showed that the stillbirth rate in pre-detected FGR was much lower than in undetected cases (FGR detected before birth, 9.7% vs. not detected before birth, 18.9%) [17]. However, because FGR is caused by multiple factors, inaccuracies in early detection still occur [18]. Particularly, late FGR often presents with a low degree of placental disease and a normal umbilical Doppler index [19]. Late FGR are 70–80% of all FGR cases, and early detection helps determine the follow-up interval or optimal delivery timing [20]. However, most cases of late-onset FGR remain undetected, often leading to emergency cesarean section, neonatal acidosis, and neonatal intensive care unit admission [14,17]. There have been various attempts to predict FGR using factors such as maternal characteristics, mean arterial pressure, and ultrasound and biochemical markers [11,21,22,23,24]. Although these models have improved the prediction of early-onset FGR, they remain unsatisfactory in identifying late-onset FGR. Moreover, the appropriate settings for clinical use remain insufficient [25].

Therefore, this study aimed to develop a prediction model for late FGR using routinely gathered variables during antenatal care.

## 2. Materials and Methods

### 2.1. Data Source

This retrospective study was conducted based on medical records obtained from seven secondary and tertiary hospitals under the College of Medicine, Catholic University of Korea, from January 2009 to December 2020. Baseline characteristics (maternal demographics, underlying disease, and social and family histories) and clinical characteristics (laboratory data, ultrasonographic finding, blood pressure, height, and weight) were obtained from medical records. The institutional review boards of the Catholic University of Korea approved this study (XC20WIDI0103). Because of the retrospective nature of this study, the requirement for informed consent was waived.

### 2.2. Definitions of FGR

FGR was defined as less than 10% birth weight, taking into account gestational age; the rest were assigned to the control group. The reference values of birth weight percentile according to gestational age were based on the Korean birth weight chart [26]. Late FGR was defined as FGR with deliveries at ≥34 weeks of gestation [27]. Multiple pregnancies, maternal age less than 18 years at delivery, delivery before 24 weeks of gestation, pregnancy with fetal chromosomal abnormalities and major congenital anomalies, and early FGR cases resulting in delivery before 34 weeks of gestation were excluded. Two obstetricians reviewed the charts to verify the data and eliminate the missing data (J.H.W. and H.S.K.).

### 2.3. Machine Learning Analysis

#### 2.3.1. Data Preparation

We followed the guidelines of the Transparent Reporting of a multivariable prediction model for Individual Prognosis or Diagnosis statement to establish the prediction models [28]. Following these guidelines, all de-identified data from 32,301 participants were included in the dataset used in this study. We divided the participants into two groups based on their obstetric history: nulliparous (*n* = 16,660) and multiparous (*n* = 15,641). To ensure an equitable distribution of the target variable, we performed a 7:3 stratification to divide the data for each of the three groups (whole, nulliparity, and multiparity) into training and test sets. Each training and test data was divided into FGR and non-FGR groups.

#### 2.3.2. Variables Used to Develop the Late FGR Prediction Models

Two sets of variables were obtained from two gestational periods and used to develop the FGR prediction models: (1) first trimester (E1) variables collected until 13 weeks of gestation and (2) early third trimester (T1) variables collected until 28 weeks of gestation. These variables contained age, parity, underlying diseases, family history, reproductive history, physical examination results, laboratory results, and obstetric history of previous pregnancies. All sets contained baseline characteristics and physical examinations, including 140 variables for nulliparous women and 170 variables for multiparous women. The E1 set contained human chorionic gonadotropin, pregnancy-associated plasma protein-A, and nuchal translucency through ultrasound examination such as Down screening tests, resulting in 178 variables in nulliparous women and 208 variables in multiparous women. The T1 set contained 362 and 398 variables for nulliparous and multiparous women, respectively. The whole cohort used the same sets of variables at E1 and T1 time points as those used in the multiparous cohort.

#### 2.3.3. Machine Learning Algorithm and Interpretation

An extreme gradient boosting machine (XGBM) algorithm was used as the machine learning method. The split dataset was input into the algorithm, and its performance was assessed using the area under the receiver operating characteristic curve (AUC) and the area under the precision-recall curve (AUPR). To represent the impact of each variable on the model output, we utilized XGBoost’s embedded feature selection method, specifically using the Gain approach to identify importance variables [29]. Gain measures the improvement in model performance brought by each variable and allows for a more targeted selection of impactful features. Related visualizations are included to highlight the contribution of these selected features to the model’s performance.

#### 2.3.4. Evaluation and Validation of the Simplified Model

For clinical applications, a simplified model was developed using up to 15 variables selected through XGBoost’s embedded feature selection with the Gain approach. The Gain method allowed us to identify the variables that most significantly contributed to model accuracy, making it a suitable choice for creating a streamlined version of the model. The performance of the simplified model was assessed using AUC and AUPR and validated using the test sets. Based on the performance and convenience of the model, a clinically applicable questionnaire was developed.

## 3. Results

### 3.1. Data Set

**Figure 1:**
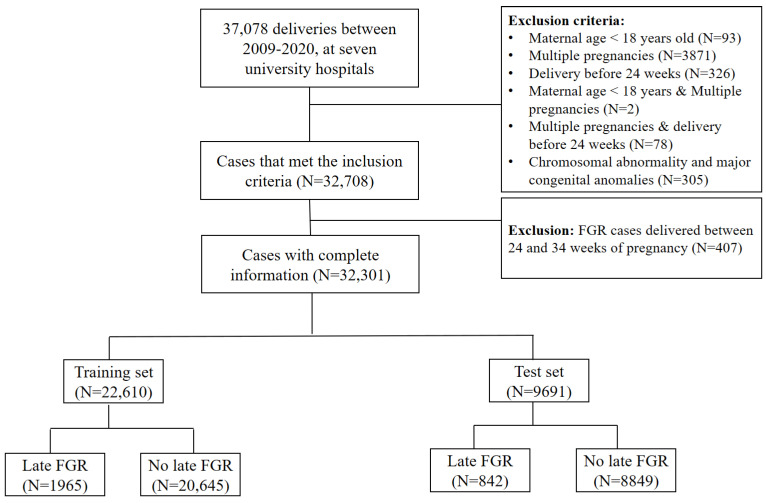
Study flow chart. FGR, fetal growth restriction.

After applying the exclusion criteria to 37,078 pregnancies, the final cohort that met the eligibility criteria included 32,301 women (Figure 1). Late FGR was diagnosed in 2807 pregnancies (8.69%) in the entire cohort, with 1965 (8.69%) and 842 (8.69%) pregnancies in the training and test sets, respectively. The baseline characteristics of the patients are described in Table 1.

### 3.2. Machine Learning Predictive Models for Late FGR at E1 and T1 Periods

**Figure 2:**
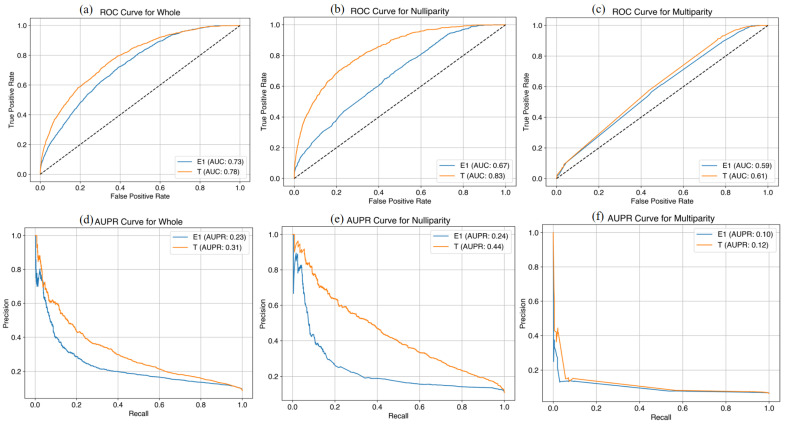
Machine learning predictive models at E1 and T1 time points with original variables for late FGR (training set). (**a**) AUC curves in the whole cohort; (**b**) AUC curves in the nulliparous cohort; (**c**) AUC curves in the multiparous cohort; (**d**) AUPR curves in the whole cohort; (**e**) AUPR curves in the nulliparous cohort; (**f**) AUPR curves in the multiparous cohort. FGR, fetal growth restriction; AUC, area under the curve; AUPR, area under the precision-recall curve.

**Figure 3:**
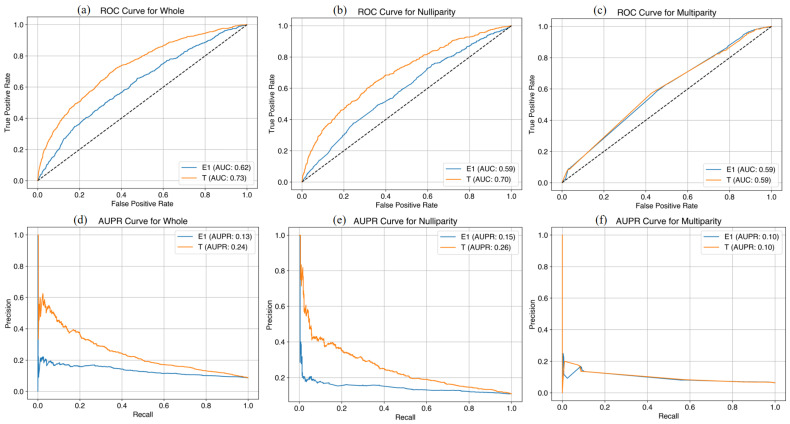
Machine learning predictive models at E1 and T1 time points with original variables for late FGR (test set). (**a**) AUC curves in the whole cohort; (**b**) AUC curves in the nulliparous cohort; (**c**) AUC curves in the multiparous cohort; (**d**) AUPR curves in the whole cohort; (**e**) AUPR curves in the nulliparous cohort; (**f**) AUPR curves in the multiparous cohort. FGR, fetal growth restriction; AUC, area under the curve; AUPR, area under the precision-recall curve.

In the prediction model using the original variables for the whole cohort, the AUC was 0.73 at E1 and 0.78 at T1 and the AUPR was 0.23 at E1 and 0.31 at T1 in the training set (Figure 2), while the AUC was 0.62 at E1 and 0.73 at T1 and the AUPR was 0.13 at E1 and 0.24 at T1 in the test set (Figure 3). In the prediction model using the original variables for nulliparous cohort, the AUC was 0.67 at E1 and 0.83 at T1 and the AUPR was 0.24 at E1 and 0.44 at T1 in the training set, while the AUC was 0.59 at E1 and 0.70 at T1 and the AUPR was 0.15 at E1, and 0.26 at T1 in the test set. In the prediction model using original variables for multiparous cohort, the AUC was 0.59 at E1 and 0.61 at T1 and the AUPR was 0.10 at E1 and 0.12 at T1 in the training set, while the AUC was 0.59 at E1 and 0.59 at T1 and the AUPR was 0.10 at E1 and 0.10 at T1 in the test set. We added the performance of the machine learning predictive model in Supplementary Table S1.

### 3.3. Feature Importance for Late FGR in the Prediction Models

**Figure 4:**
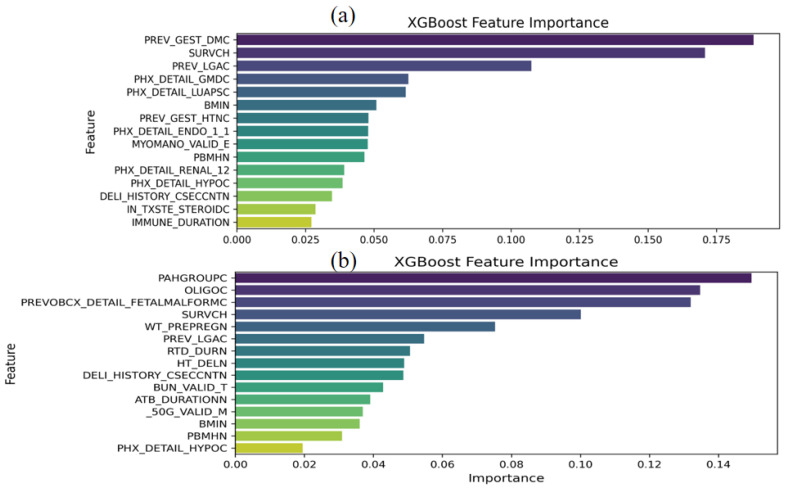
Feature importance for late FGR with XGBoost’s embedded feature selection method. (**a**) E1 and (**b**) T1. PREV_GEST_DMC, previous gestational diabetes mellitus; SURVCH, parity; PREV_LGAC; previous LGA; PHX_DETAIL_LUAPSC, history of lupus or antiphospholipid syndrome; BMIN, pre-pregnancy body mass index; PREV_GEST_HTNC, previous pregnancy associated hypertension; PHX_DETAIL_ENDO_1_1/history of hyperthyroidism; MYOMANO_VALID_E, number of uterine myoma; PBMHN, number of preterm birth; PHX_DETAIL_RENAL_12, history of renal disease; PHX_DETAIL_HYPOC, history of hypothyroidism; DELI_HISTORY_CSECCNTN, number of previous cesarean section; IN_TXSTE_STEROIDC; use of any steroid treatment; IMMUNE_DURATION, duration of immune disease; PAHGROUPC, pregnancy associated hypertension at current pregnancy; OLIGOC, oligohydramnios; PREVOBCX_DETAIL_FETALMALFORMC, previous fetal malformation; WT_PREPREGN, pre-pregnancy weight; RTD_DURN, duration of ritodrine; HT_DELN, height; BUN_VALID_T, blood urea nitrogen at T1 period; ATB_DURATION, atosiban duration; _50G_VALID_M, 50 g glucose challenge test value at midtrimester.

The feature importance for late FGR with XGBoost’s embedded feature selection method up to 15 variables in the entire cohort is presented in Figure 4. In the whole cohort, previous gestational diabetes mellitus (GDM), parity, previous large for gestational age (LGA), pre-pregnancy body mass index (BMI), previous pregnancy associated hypertension (PAH), number of preterm birth, number of cesarean section, pre-existing disease (renal and glomerular disease, lupus or antiphospholipid syndrome, impaired glucose tolerance, thyroid disease, immune disease), myoma, and use of any steroid treatment were selected as the most predictive features at E1. At T1, the variables selected as the most predictive features were PAH at current pregnancy, oligohydramnios, previous fetal malformation, parity, pre-pregnancy weight, previous LGA, duration of tocolytics (ritodrine and atosiban), maternal height, number of cesarean section, blood urea nitrogen (BUN) at T1 period, 50 g glucose challenge test (GCT) value at mid trimester, pre-pregnancy BMI, number of preterm birth, and hypothyroidism.

### 3.4. Feature Selections for a Simplified Prediction Model of Late FGR and Performances

**Figure 5:**
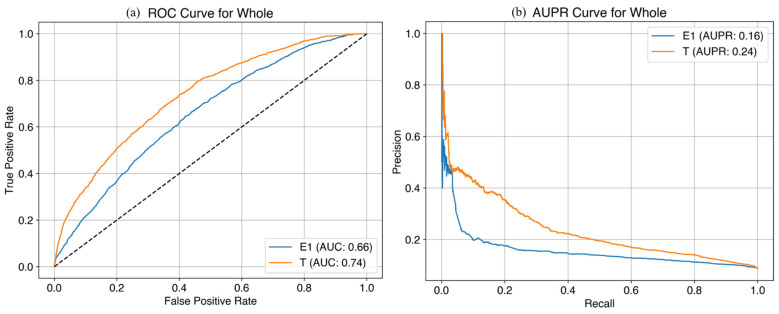
Performance of the simplified prediction model at E1 and T1 time points for late FGR in the entire cohort (training set). (**a**) AUC curves; (**b**) AUPR curves. FGR, fetal growth restriction; AUC, area under the curve; AUPR, area under the precision-recall curve.

**Figure 6:**
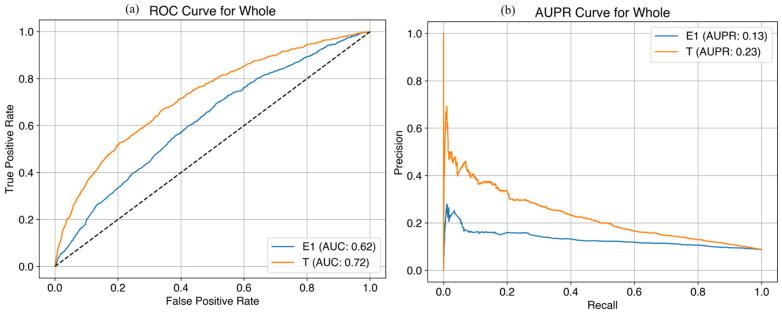
Performance of the simplified prediction model at E1 and T1 time points for late FGR in the entire cohort (test set). (**a**) AUC curves; (**b**) AUPR curves. FGR, fetal growth restriction; AUC, area under the curve; AUPR, area under the precision-recall curve.

Consequently, we developed a simplified questionnaire using E1 and T1 variables with high importance for clinical use (Table 2). In the simplified prediction model for late FGR based on the whole cohort, the AUC was 0.66 at E1 and 0.74 at T1 and the AUPR was 0.16 at E1 and 0.24 at T1 in the training set (Figure 5); the AUC was 0.62 at E1 and 0.72 at T1 and the AUPR was 0.13 at E1 and 0.23 at T1 in the test set (Figure 6). We added the performance of prediction model with high importance in Supplementary Table S2.

## 4. Discussion

We developed a machine learning model to predict late FGR using variables at two gestational periods. Predictive performance becomes more accurate as pregnancy progresses and more clinical data become available. The performance of the simplified prediction model using important variables with XGBoost’s embedded feature selection was similar to that of the original model.

The most important predictive variables for late FGR were previous GDM, parity, previous LGA, maternal glomerular disease at E1, and PAH at current pregnancy, oligohydramnios, previous fetal malformation, and parity at T1.

Early FGR is often classified as a high-risk pregnancy and is often accompanied by Doppler abnormalities or maternal pre-eclampsia and managed intensively; however, late FGR often goes undetected [12,13].

A previous prospective cohort study found that the continuous evaluation of fetal growth from the second to the third trimester had poor ability to predict late FGR in low-risk singleton pregnancies [30]. Another recent prospective study recently developed a predive model for FGR in the first trimester and found comparable prediction performance between early and late FGR (AUC of early FGR 0.77 vs. AUC of late FGR 0.79) [31]. However, the variables used in their prediction model included the uterine artery and ductus venosus pulsatility index, several biomarkers which are not routinely checked in low-risk maternal care.

Previous studies have often screened for FGR using factors associated with pre-eclampsia [32,33]. In this study, previous PAH at E1 and PAT at current pregnancy at T1 were also included in the prediction model. We found that maternal height, pre-pregnancy BMI, number of previous cesarean sections, parity, and myoma were predictive factors for late FGR, which is consistent with previous literature [34,35,36,37]. However, it is unclear whether a previous cesarean section is simply associated with FGR or whether it is itself a cause.

In this study, previous GDM and previous LGA were important features for subsequent FGR. Recent study found that previous GDM is an independent risk factor for GDM, PAH, and LGA in subsequent pregnancy [38]. Previous studies also have shown that previous FGR is the strongest predictor of FGR and previous LGA is the strongest predictor of LGA [39,40]. Previous LGA may be features with negative correlation with FGR, although feature importance in the model does not provide information about negative or positive direction, as other machine learning studies had not shown [39].

The association between previous preterm birth and subsequent FGR has been revealed not only in this study but also in a previous study [41].

Our findings also indicated that the duration of ritodrine and atosiban administration were important features. Previous study reported that β2 adrenoceptors (β2AR) play a key role via rapamycin complexes 1 and 2 (mTORC1/2) in the regulation of skeletal muscle glucose oxidation in models of intrauterine growth restriction [42]. Adrenergic adaptation is associated with unsatisfactory glucose oxidation, which is known to be a characteristic of skeletal muscles in the FGR group. They suggested that β2 adrenergic enhancement could be a therapeutic target for FGR-mediated metabolic dysfunction. This shows that the use of ritodrine is inversely related to FGR. But the relationship between atosiban and FGR has not yet been clearly studied. Although those variables selected through XGBoost’s embedded feature selection method showed some correlation to FGR, it does not mean that those have causal relationship with FGR.

Maternal serum BUN levels were included in the simplified model. Gestational renal dysfunction may lead to maladaptation to normal physical conditions during pregnancy. Several studies have also found that elevated levels of BUN, uric acid, Cr, and cystatin C are associated with the development of FGR [43,44]. Maternal pre-existing diseases were also shown to be important features affecting FGR, which is consistent with previous studies [45,46,47].

This study had several limitations. First, this study has retrospective nature, which led us to define FGR based on neonatal birth weight (below the 10th percentile) and include several missing data and biases. However, because the seven hospitals in this study used the same electronic medical record (EMR) form, the bias in information from the EMR was relatively low. Second, the number of patients with FGR was lower than that in the control group. Nevertheless, considering that the prevalence of FGR is 7–10% of all pregnancies, the number of FGR cases included in this study is appropriate [22]. Despite hyperparameter tuning beyond scale_pos_weight to maximize the stability and performance of the model, we acknowledge that class imbalance continues to impact classification performance for the patient group. In future research, we plan to validate this model in a prospective cohort and try to enhance model performance by exploring additional approaches to increase data diversity and address the imbalance problem. However, it seems that low values of AUPR, compared to the AUC levels, might be attributed to data imbalance. Third, birth weight < 10% rather than EFW < 10% or AC < 10% was used as the diagnostic criterion for FGR [26]. 

The first strength of this study is that we utilized data from seven centers located in different regions which reflected various characteristics. Second, we obtained an AUC of 0.62 at E1 and 0.73 at T1 and an AUPR of 0.13 at E1 and 0.24 at T1. The specificity was 0.82 at E1 and 0.89 at T1, and the sensitivity was 0.35 at E1 and 0.38 at T1. In a recent study on predicting late FGR using machine learning, the Random Forest algorithm was used, and the AUC was 0.81 and the positive predictive value was 73% [48]. In another prospective study, the AUC was 0.79 with a specificity of 0.78 and a sensitivity of 0.69 for late FGR [31].

The model used in this study showed similar performance and, unlike previous studies, has the advantage of being highly usable by using markers routinely measured in clinical care.

Therefore, the simplified prediction model based on data collected during routine antenatal care may be able to estimate the individual risk of late FGR and closely monitor women at high risk.

## 5. Conclusions

The simplified machine learning model for predicting late FGR may be useful for evaluating individual risks in the early third trimester. Future large prospective studies to verify this prediction model may be needed.

## Figures and Tables

**Table 1:** Baseline characteristics of patients with late FGR.

Variables	Training Set (N = 22,610)			Test Set (N = 9691)		
	No Late FGR ^a^ (N = 20,645)	Late FGR (N = 1965)	*p*-Value	No Late FGR (N = 8849)	Late FGR (N = 842)	*p*-Value
Age						
Mean (SD)	32.77 (4.46)	32.27 (4.69)	<0.001	32.88 (4.43)	32.19 (4.61)	<0.001
<35 years, n (%)	13,459 (65.19)		0.001	5712 (64.55)	580 (68.88)	0.013
≥35 years, <40 years, n (%)	5938 (28.76)	490 (24.94)		2558 (28.91)	224 (26.6)	
≥40 years, n (%)	1248 (6.05)	115 (5.85)		579 (6.54)	38 (4.51)	
Nulliparity, n (%)	10,422 (50.48)	1268 (64.53)	<0.001	4426 (50.02)	544 (64.61)	<0.001
Maternal height (cm), mean (SD)	161.44 (5.34)	160.09 (5.36)	<0.001	161.46 (5.24)	159.88 (5.28)	<0.001
BMI ^a^ before pregnancy, kg/m^2^						
Mean (SD)	21.83 (3.72)	20.98 (3.29)	<0.001	21.84 (3.78)	21.10 (3.52)	<0.001
<25 kg/m^2^, n (%)	17,303 (84.18)	1756 (89.73)		7437 (84.36)	735 (87.60)	0.029
≥25 kg/m^2^, <30 kg/m^2^, n (%)	2445 (11.90)	156 (7.97)		1009 (11.45)	81 (9.65)	
≥30 kg/m^2^, n (%)	806 (3.92)	45 (2.30)		370 (4.20)	23 (2.74)	
BMI at delivery, kg/m^2^						
Mean (SD)	26.59 (3.93)	25.52 (3.65)	<0.001	26.60 (4.02)	25.56 (3.74)	<0.001
<25 kg/m^2^, n (%)	7857 (38.15)	971 (49.59)	<0.001	3386 (38.37)	412 (49.11)	<0.001
≥25 kg/m^2^, <30 kg/m^2^, n (%)	9357 (45.44)	794 (40.55)		4000 (45.33)	336 (40.05)	
≥30 kg/m^2^, n (%)	3380 (16.41)	193 (9.86)		1439 (16.31)	91 (10.85)	
Weight gain during pregnancy, mean (SD)	12.39 (5.11)	11.62 (4.80)	<0.001	12.40 (5.13)	11.38 (4.67)	<0.001
Preexisting disease						
Hypertension, n (%)	760 (3.68)	88 (4.48)	0.076	352 (3.98)	46 (5.46)	0.038
Diabetes, n (%)	254 (1.23)	17 (0.87)	0.155	90 (1.02)	4 (0.48)	0.125
CKD ^a^, n (%)	46 (0.22)	10 (0.51)	0.027	21 (0.24)	4 (0.48)	0.270
Arrhythmia, n (%)	291 (1.41)	10 (0.51)	0.001	128 (1.45)	13 (1.54)	0.822
Renal disease, n (%)	194 (0.94)	34 (1.73)	0.001	82 (0.93)	18 (2.14)	0.001
Lupus, n (%)	128 (0.62)	32 (1.63)	<0.001	47 (0.53)	16 (1.90)	<0.001
Hypothyroidism, n (%)	1063 (5.15)	78 (3.97)	0.023	441 (4.98)	32 (3.80)	0.128
Pregnancy complication						
GDM ^a^, n (%)	1612 (7.81)	124 (6.31)	0.017	743 (8.40)	50 (5.94)	0.013
Gestational hypertension, n (%)	399 (1.93)	86 (4.38)	<0.001	149 (1.68)	36 (4.28)	<0.001
Preeclampsia, n (%)	665 (3.22)	233 (11.86)	<0.001	294 (3.32)	104 (12.35)	<0.001
Eclampsia, n (%)	16 (0.08)	4 (0.20)	0.09	3 (0.03)	2 (0.24)	0.063
Superimposed pre-eclampsia, n (%)	130 (0.63)	27 (1.37)	<0.001	55 (0.62)	9 (1.07)	0.126
Previous pregnancy history						
Previous preterm delivery history, n (%)	1349 (6.53)	88 (4.48)	<0.001	585 (6.61)	51 (6.06)	<0.001
Previous pre-eclampsia history, n (%)	376 (1.82)	57 (2.90)	<0.001	160 (1.81)	19 (2.26)	<0.001
Previous FDIU ^a^ history, n (%)	161 (0.78)	10 (0.51)	<0.001	73 (0.82)	9 (1.07)	<0.001
Previous GDM history, n (%)	356 (1.72)	28 (1.42)	<0.001	162 (1.83)	6 (0.71)	<0.001
Previous FGR history, n (%)	390 (1.89)	78 (3.97)	<0.001	178 (2.01)	33 (3.92)	<0.001
Previous placenta previa history, n (%)	180 (0.87)	12 (0.61)	<0.001	70 (0.79)	4 (0.48)	<0.001
Previous PAS ^a^ history, n (%)	54 (0.26)	4 (0.20)	<0.001	18 (0.20)	3 (0.36)	<0.001
Previous postpartum hemorrhage history, n (%)	3491 (16.91)	183 (9.31)	<0.001	1487 (16.80)	83 (9.86)	<0.001
Myoma, n (%)	3028 (14.67)	286 (14.55)	0.893	1350 (15.26)	121 (14.37)	0.494
IVF ^a^, n (%)	542 (2.63)	45 (2.29)	0.372	222 (2.51)	19 (2.26)	0.653
Paternal age, years						
Mean (SD)	35.41 (4.87)	35.13 (4.93)	0.008	35.44 (4.80)	34.88 (4.93)	0.002
<35 years, n (%)	8728 (44.66)	886 (48.63)	0.001	3719 (44.42)	393 (50.13)	0.002
≥35 years, n (%)	10,814 (55.34)	936 (51.37)		4653 (55.58)	391 (49.87)	

Abbreviations (^a^): FGR, fetal growth restriction; BMI, body mass index; CKD, chronic kidney disease; GDM, gestational diabetes mellitus; FDIU, fetal death in the uterus; PAS, placenta accreta spectrum; IVF, in vitro fertilization.

**Table 2:** Simplified 20-point questionnaire for predicting late FGR ^a^.

**Baseline check**
Please answer:
Age: __________ years old
Height: __________ cm
Pre-pregnancy weight: ____________ kg
How many times have you given birth before? _____________
Do you have uterine myoma? Yes/No
Have you ever been diagnosed with an renal or glomerular disease? Yes/No
Have you ever been diagnosed with lupus or antiphospholipid syndrome? Yes/No
Have you ever been diagnosed with impaired glucose disease? Yes/No
Have you ever been diagnosed with hypo/hyperthyroidism? Hypo (Yes/No) Hyper (Yes/No)
Have you ever been diagnosed with immune disease? Yes (duration:___ years)/No
Have you ever been treated with steroid medication? Yes/No
Please fill out / check only if you have given birth before:
Have you ever undergone a cesarean section in a previous pregnancy? Yes/No
If yes, number of cesarean sections ______
In your last pregnancy, was your baby small or large for gestational age? SGA ^a^/LGA ^a^/No
Have you ever had a preterm birth in a previous pregnancy? Yes/No
If yes, number of preterm births ______
Have you ever had a pregnancy with a congenital anomaly? Yes/No
Have you ever been diagnosed with gestational diabetes mellitus? Yes/No
Have you ever been diagnosed with gestational hypertension or pre-eclampsia in a previous pregnancy?
Yes/No
**T: Late pregnancy variables (final results until 28 weeks of gestation)**
To be written by the clinician
Ultrasonographic abnormalities: Oligohydramnios: Yes/No
Obstetric complications:
Gestational hypertension: Yes/No, (Superimposed)Pre-eclampsia/eclampsia: Yes/No
Tocolytics during pregnancy (If no, 0):
Ritodrine ______days, Atosiban ______days, Nifedipine ____days
The last lab results
50 g GCT ^a^: _____________ mg/dL
BUN ^a^ _____ mg/dL, Cr ^a^ _______ mg/dL

Abbreviations (^a^): FGR, fetal growth restriction; SGA, small for gestational age; LGA, large for gestational age; BUN, blood urea nitrogen; Cr, creatinine; GCT, glucose challenge test.

## Data Availability

Data is unavailable due to privacy or ethical restrictions, according to decision by the Data Review Boards of The Catholic University of Korea.

## Supplementary Materials

The following supporting information can be downloaded at: https://www.mdpi.com/article/10.3390/life14111521/s1, Table S1. Performance of machine learning with original variable. Table S2. Performance of prediction model with high importance.
