# Supplementary material for: Prediction Model of Late Fetal Growth Restriction with Machine Learning Algorithms
Source: Life (Basel). 2024 Nov 20;14(11):1521. doi: 10.3390/life14111521 (PMC11595523; doi:10.3390/life14111521)
Supplement: Supplementary file 1 [file life-14-01521-s001.zip › life-3268547-supplementary.pdf]

Suppl Table 1. Performance of machine learning with original variable.

	Specificity	Sensitivity	Precision	F1
Whole at E1	0.82	0.35	0.16	0.22
Whole at T1	0.89	0.38	0.25	0.30
Nulliparity at E1	0.76	0.38	0.16	0.22
Nulliparity at T1	0.89	0.36	0.28	0.31
Multiparity at E1	0.55	0.59	0.08	0.14
Multiparity at T1	0.57	0.75	0.08	0.15

Suppl Table 2. Performance of prediction model with high importance.

	Specificity	Sensitivity	Precision	F1
Whole at E1	0.63	0.55	0.12	0.20
Whole at T1	0.9	0.38	0.25	0.30
Nulliparity at E1	0.58	0.55	0.14	0.22
Nulliparity at T1	0.87	0.38	0.27	0.31
Multiparity at E1	0.5	0.62	0.08	0.14
Multiparity at T1	0.62	0.55	0.09	0.15
